# Light-programmable manipulation of DC field in Laplacian Meta-devices

**DOI:** 10.1038/s41598-018-30612-5

**Published:** 2018-08-15

**Authors:** Tiancheng Han, Yuexia Liu, Lei Liu, Jin Qin, Ying Li, Jiayu Bao, Dongyuan Ni, Cheng-Wei Qiu

**Affiliations:** 1 School of Physical Science and Technology, Southwest University, Chongqing, 400715 China; 2 Department of Electrical and Computer Engineering, National University of Singapore, 4 Engineering Drive 3, 119620 Singapore, Republic of Singapore

## Abstract

Impressive progresses have been achieved in the field of metamaterial to mimic the illusion or camouflage effects in nature. These include invisible cloaks and many other cloak-based illusion meta-devices. However, to date many experiments only present single, static or discretized functionalities. The dynamical control of multiple kinds of illusion signals can only be achieved by embedding complex active sources directly connected to external stimuli, leading to limited on/off switching effect in a contact fashion. Here, we experimentally demonstrate a distinct scheme to incorporate multi-functions into one passive Laplacian DC meta-device, assisted by light illumination. It is shown that light-programmable cloaking, full illusion, and partial illusion can be achieved on the same device without physical contact of the heating pads or electric bias, at the cost of only four kinds of natural bulk materials with homogeneous parameters throughout. A DC network is fabricated to demonstrate the proof of concept, with measurement results in good agreement with the numerical simulations. The proposed scheme may open a new avenue to the non-contact multiphysical control of multi-illusion functions for Laplacian fields.

## Introduction

In nature, many creatures have mastered the skill of rendering their appearances, for various purposes from camouflage to courtship. The most powerful and fascinating one might be the ability to generate illusions, such that a creature will be perceived as something else or nothing. Recently, great efforts have been made to realize the analogue of illusion effects in artificial metamaterials or meta-devices, resulting in the prominent achievements of invisible cloaks^1–3^ and many other cloak-based illusion devices^4–6^. Thanks to the pioneering theoretical works of transformation optics (TO) and scattering cancellation (SC) technology^7–9^, these devices are able to manipulate the signal of an arbitrary object with great freedom and high accuracy.

**Figure 1:**
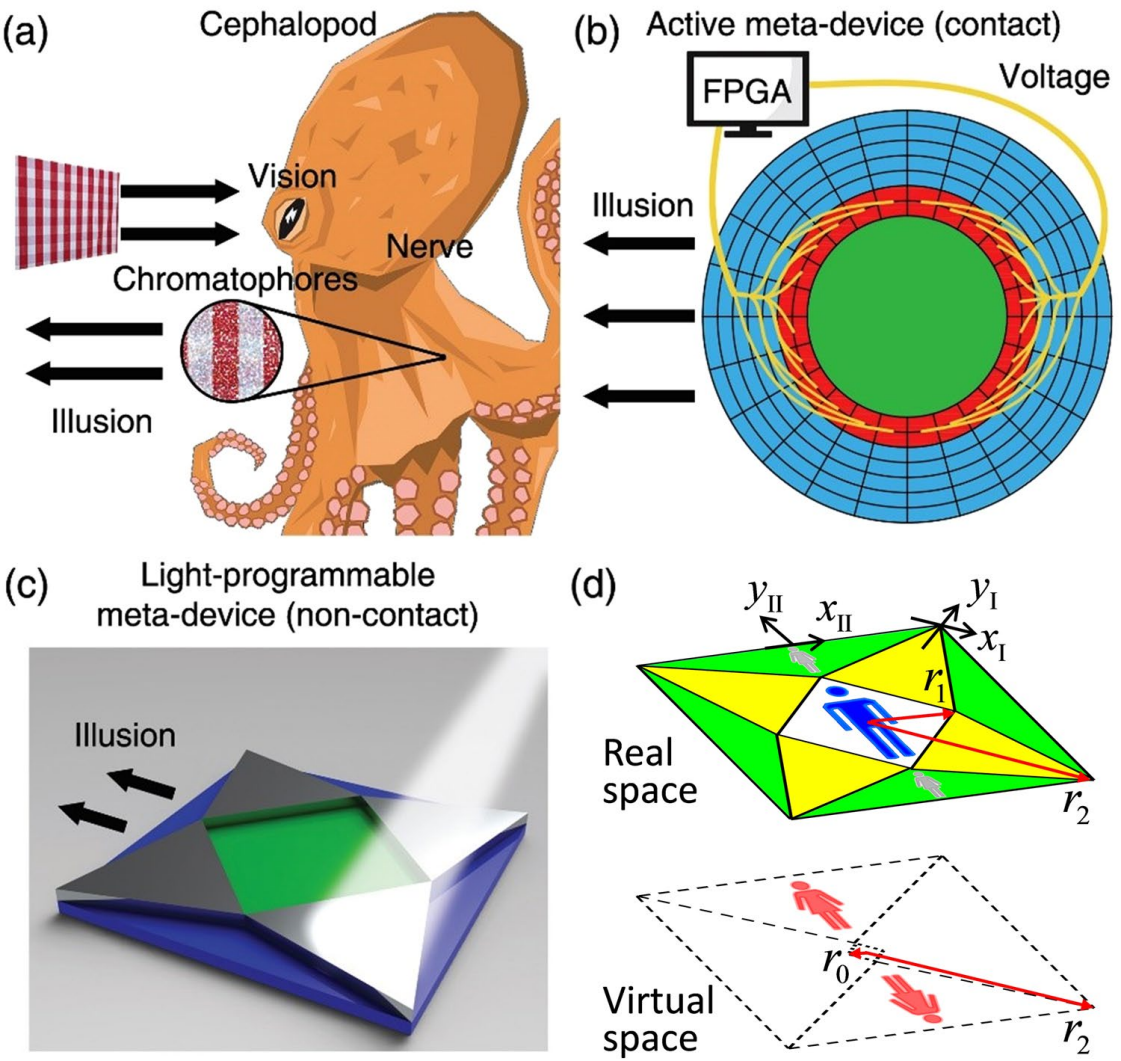
(**a**) The skin texture and color pattern of cephalopods can be fully controlled to mimic their surroundings, showing smart camouflage in nature. (**b**) A typical switchable illusion device for Laplace field, whose active modules (red colored) are connected with and controlled by a FPGA. (**c**) Schematic demonstration of the proposed light-programmable illusion meta-device. (**d**) The schematic of coordinate transformation in which a “man” shrouded by the proposed meta-device will be perceived as two “woman”.

However, the current illusion meta-devices are still far from being able to fully imitate the performances in nature. One of their most significant limitations is that these meta-devices cannot easily adjust its illusion signals based on different circumstances, which is an essential function of many animals. A typical example can be found in cephalopods, whose skin texture and color pattern can be fully controlled to mimic their surroundings. The entire process is schematically plotted in Fig. 1(a). When encountering a new environment, an octopus first gathers information with its vision. Then controlled by its nervous system, the color pattern and texture of its skin is changed to match this environment through cells such as chromophores, thereby sending out illusive signals.

For previous illusive meta-devices, replication of the above processes is very difficult. On the one hand, the realization of a normal meta-device such as cloak is already challenging because of the extreme constitutive parameters (inhomogeneous, anisotropic, and even singular)^10–12^, even for carpet cloaks that attempt to hide an object on the ground and mimic a half-infinite vacuum space^13–18^. On the other hand, to achieve an *in-situ* control over the rendered illusion, some active sources must be embedded in the system, which are usually tuned through direct connection to external terminals^19,20^. As illustrated in Fig. 1(b), the active modules (red colored) in a Laplacian meta-device are connected with and controlled by a Field-Programmable Gate Array (FPGA). The complexity and inconvenience of such a system are obvious. Moreover, most experimentally realized devices can only be switched on and off between two states, say a cloaking and a non-cloaking state^21–23^.

Here, we propose an illusion meta-device for Laplacian fields which is much simpler in configuration and programmable through non-contact operations. Compared with traditional practices for TO devices which requires a large number of elements and gives up theoretical exactness based on effective-medium approximation (EMA)^21–23^, our device consists of eight components that are homogeneous without any singularity or approximation. Each component can be actively controlled in a non-contact way with light, as schematically show in Fig. 1(c). As a simple demonstration, we experimentally realized the meta-device using a resistor-network, with only eight kinds of resistors. The results are in excellent agreement with numerical simulations, and show a multifunction of cloak, full illusion, and partial illusion that is tunable through light illumination. Our work implies an efficient approach towards the remote control of illusion meta-device that is readily applicable to all kinds of Laplacian fields, including DC magnetic field^24–27^ and thermal field^28–35^.

## Results

### Theoretical modeling

We start with general redirection and control of steady current fields. In a source-free conductive region, the currents are governed by $\nabla \cdot \overrightarrow{j}=0$. Since $\overrightarrow{j}=\sigma \overrightarrow{E}$ and $\overrightarrow{E}=-\,\nabla V$, the electric potential *V* satisfies the Laplace equation $\nabla \cdot (\sigma \nabla V)=0$. We know that *V* is uniquely determined by σ when the boundary conditions are fixed. It means that the potential distributions (as well as currents) can be manipulated at will by properly designing the conductivity of the medium without changing the boundary conditions. Due to the form invariance of Laplace equation, coordinate transformation provides a theoretical tool for us to achieve this destination. After the coordinate transformation, the electric potential *V*′ always satisfies $\nabla \cdot (\sigma ^{\prime} \,\nabla V^{\prime} )=0$, where *V*′ and σ**′** are the electric potential and conductivity in the new space, respectively. We assume that the original space and new space are denoted as (*x, y, z*) and (x′, y′, z′), respectively, the transformed conductivity of the medium can be obtained as $\overrightarrow{\sigma ^{\prime} }=\sigma {\bf{A}}{{\bf{A}}}^{{\rm{T}}}/|{\bf{A}}|$, where ${\bf{A}}=\frac{\partial (x^{\prime} ,y^{\prime} ,z^{\prime} )}{\partial (x,y,z)}$ is the Jacobian transformation matrix.

The coordinate transformation of the proposed meta-device is schematically illustrated in Fig. 1(d), which can be derived based on a two-fold operation. The first is to eliminate the scattering of the original object by using a DC cloak, and the second is to create some convertible and tunable illusion objects in virtual space. To avoid inhomogeneity and singularity in previous invisibility cloaks^21–23^, we propose a square DC cloak based on the linear homogenous coordinate transformation. The square DC cloak is achieved by stretching the small square with radius *r*_0_ into a bigger square with radius *r*_1_, while the outer square with radius *r*_2_ is maintained. We can see that the as-designed meta-device consists of eight triangular elements, which can be grouped into Element I (four yellow triangular) and Element II (four green triangular).

For Element I, a linear transformation is carried out in local coordinate system (*x*_I_, *y*_I_), and can be expressed as ${x^{\prime} }_{{\rm{I}}}=\frac{{r}_{1}}{{r}_{0}}{x}_{{\rm{I}}}$ and ${y^{\prime} }_{{\rm{I}}}=\frac{2{r}_{2}-\sqrt{2}{r}_{1}}{2{r}_{2}-\sqrt{2}{r}_{0}}{y}_{{\rm{I}}}$. Then the conductivities for Element I are obtained ${\sigma ^{\prime} }_{{x}_{{\rm{I}}}}=\frac{{r}_{1}(2{r}_{2}-\sqrt{2}{r}_{0})}{{r}_{0}(2{r}_{2}-\sqrt{2}{r}_{1})}{\sigma }_{0}$ and ${\sigma ^{\prime} }_{{y}_{{\rm{I}}}}=\frac{{r}_{0}(2{r}_{2}-\sqrt{2}{r}_{1})}{{r}_{1}(2{r}_{2}-\sqrt{2}{r}_{0})}{\sigma }_{0}$, where σ_0_ is the conductivity of the background material. For Element II, a linear transformation is carried out in local coordinate system (*x*_II_, *y*_II_), and can be expressed as ${x^{\prime} }_{{\rm{II}}}={x}_{{\rm{II}}}$ and ${y^{\prime} }_{{\rm{II}}}=\frac{\sqrt{2}{r}_{2}-2{r}_{1}}{\sqrt{2}{r}_{2}-2{r}_{0}}{y}_{{\rm{II}}}$. Then the conductivities for Element II are also obtained as ${\sigma ^{\prime} }_{{x}_{{\rm{II}}}}=\frac{2{r}_{0}-\sqrt{2}{r}_{2}}{2{r}_{1}-\sqrt{2}{r}_{2}}{\sigma }_{0}$ and ${\sigma ^{\prime} }_{{y}_{{\rm{II}}}}=\frac{2{r}_{1}-\sqrt{2}{r}_{2}}{2{r}_{0}-\sqrt{2}{r}_{2}}{\sigma }_{0}$. We can see that the as-designed meta-device is composed of homogeneous and anisotropic materials. No extreme values (singularity) of the material parameters are involved.

We next emulate the proposed meta-device by the circuit theory. The two resistances for the layers A can be expressed as ${R}_{{\rm{A}}i}^{x}=\frac{{\rm{\Delta }}{x}_{i}}{{\sigma }_{Ai}\cdot {\rm{\Delta }}{y}_{i}\cdot h}$ and ${R}_{{\rm{A}}i}^{y}=\frac{{\rm{\Delta }}{y}_{i}}{{\sigma }_{Ai}\cdot {\rm{\Delta }}{x}_{i}\cdot h}$ (*i* = I, II), where ${\rm{\Delta }}{x}_{i}$ and ${\rm{\Delta }}{y}_{i}$ are step lengths in the *x*_*i*_-axis and *y*_*i*_-axis, respectively. Similarly, the two resistances for the layers B can be expressed as ${R}_{{\rm{B}}i}^{x}=\frac{{\rm{\Delta }}{x}_{i}}{{\sigma }_{Bi}\cdot {\rm{\Delta }}{y}_{i}\cdot h}$ and ${R}_{{\rm{B}}i}^{y}=\frac{{\rm{\Delta }}{y}_{i}}{{\sigma }_{Bi}\cdot {\rm{\Delta }}{x}_{i}\cdot h}$ (*i* = I, II). Obviously, only four types of resistors are required when ${\rm{\Delta }}{x}_{i}={\rm{\Delta }}{y}_{i}$, and eight types of resistors are required when ${\rm{\Delta }}{x}_{i}\ne {\rm{\Delta }}{y}_{i}$.

We further extend DC cloaking to DC illusion. By mapping the illusion objects in virtual space to the corresponding region in physical space, an illusion device can be created to transform an actual perception into arbitrarily pre-controlled perception. Assuming that the illusion objects are located in the region of Element II, the material properties of the illusion region in DC device are described as ${\sigma ^{\prime} }_{{x}_{{\rm{II}}}}=\frac{2{r}_{0}-\sqrt{2}{r}_{2}}{2{r}_{1}-\sqrt{2}{r}_{2}}{\sigma }_{virtual}$ and ${\sigma ^{\prime} }_{{y}_{{\rm{II}}}}=\frac{2{r}_{1}-\sqrt{2}{r}_{2}}{2{r}_{0}-\sqrt{2}{r}_{2}}{\sigma }_{virtual}$, where σ_*virtual*_ denotes the conductivity of the illusion objects in virtual space. The material parameters of illusion region can also be realized using resistor network. Inspired by the pioneering work^23^, we add a light-dependent resistor in parallel to the original resistors to generate a non-contact light-controlled meta-device. Then the resulted resistors will be ${R^{\prime} }_{{\rm{A}}}=\frac{{R}_{{\rm{A}}}\cdot {R}_{L}}{{R}_{{\rm{A}}}+{R}_{L}}$ and ${R^{\prime} }_{{\rm{B}}}=\frac{{R}_{{\rm{B}}}\cdot {R}_{L}}{{R}_{{\rm{B}}}+{R}_{L}}$, where *R*_*L*_ is the value of the light-dependent resistor, which is controlled by the intensity of the illuminating light in a non-touching manner. Therefore, the functionality of the device is controllable with the change of illuminating light.

### Simulation results

**Figure 2:**
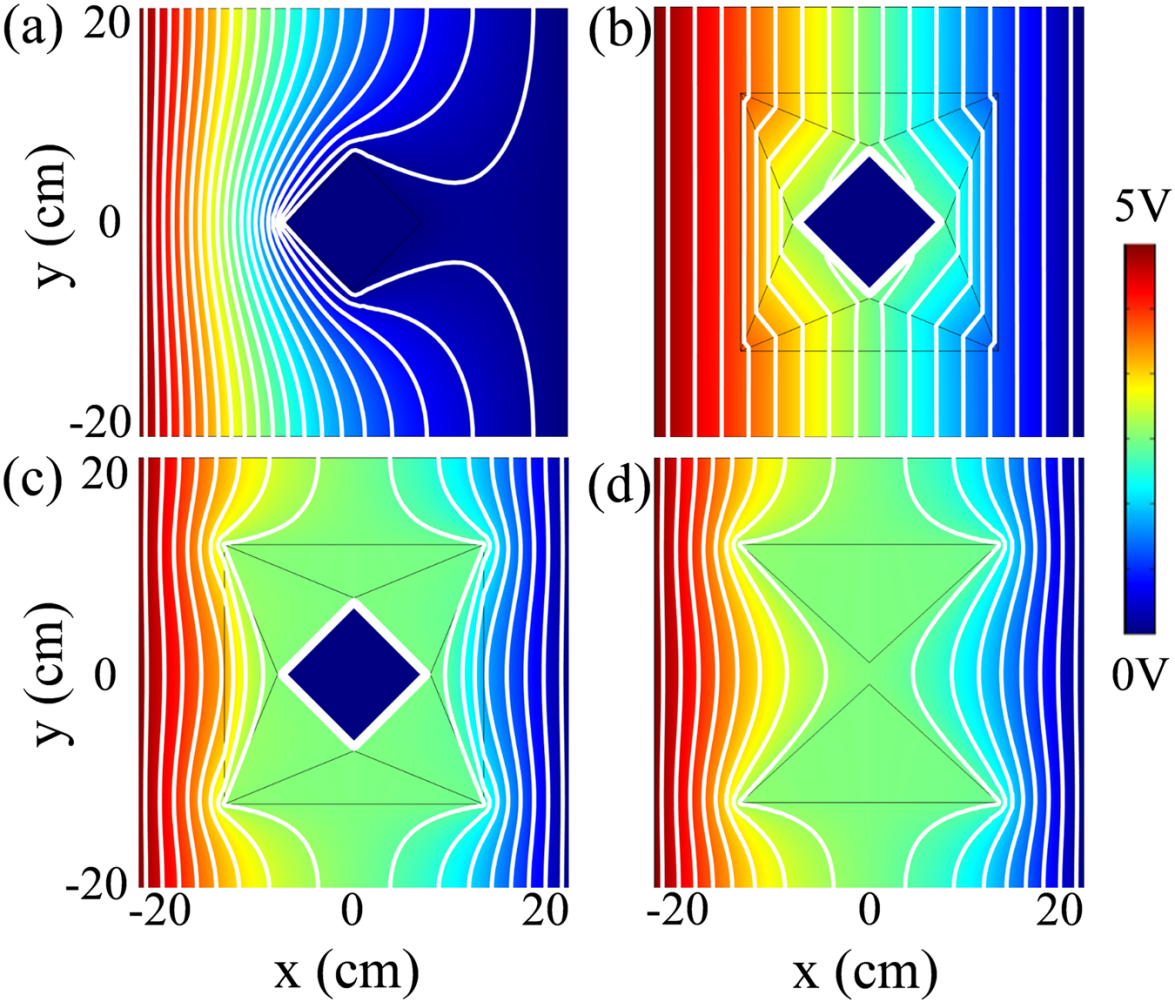
Simulated voltage distributions based on FEM in different scenarios. (**a**) A bare object (a square copper) is connected to ground. (**b**) The object is shrouded by the proposed meta-device that acts as an invisibility cloak. (**c**) The object is shrouded by the proposed meta-device that acts as an illusion device. (**d**) Two equivalent triangular insulators in virtually homogeneous space. Equipotential lines are represented with white color in the panel.

When a square copper defect is placed in the background and connected to ground, the voltage distribution is demonstrated in Fig. 2(a). As expected, the equipotential lines are significantly distorted, thus making the object visible. When the object is shrouded by the proposed meta-device, the scattering signature can be manipulated at will. In one case as shown in Fig. 2(b), the device acts as an invisibility cloak, which can be achieved by making the photoresistor value *R*_*L*_ infinite (${R^{\prime} }_{{\rm{A}}}={R}_{{\rm{A}}}$). Although the device possesses sharp corners, the equipotential lines are smoothly bent around the cloaking region and eventually return to their original pathway, which renders the central region invisible to the outside observer. In the other case as shown in Fig. 2(c), the meta-device acts as an illusion device, which can be achieved by making the photoresistor value *R*_*L*_ small (${R^{\prime} }_{{\rm{A}}}\ll {R}_{{\rm{A}}}$). To verify the illusion effect of the device, we plotted the potential distribution of the equivalent two virtual triangular insulators, as shown in Fig. 2(d). It is obvious that the equipotential lines outside the illusion device are the same in Fig. 2(c,d).

### Experimental verification

**Figure 3:**
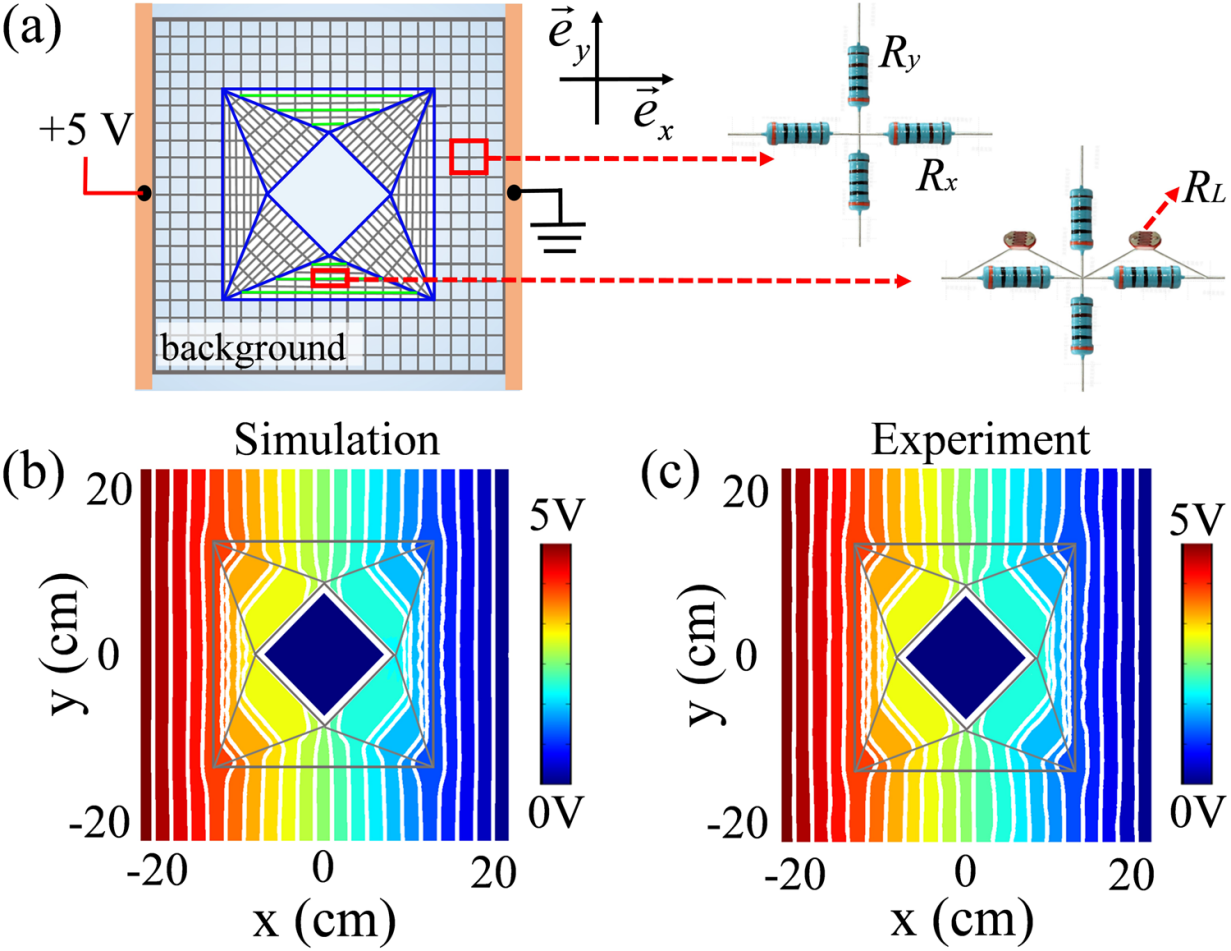
(**a**) Schematic illustration for experimental realization of the proposed light-programmable illusion meta-device. The inset is the detail of chip resistors. (**b**) Simulated voltage distribution for the reference DC cloak with the central region connected to ground. (**c**) Measured voltage distribution corresponding to (b). Equipotential lines are represented with white color in the panel.

To validate the performance of light-controlled meta-device, we fabricated a DC network to demonstrate the proof-of-concept experiment. Figure 3(a) shows the actual discretized grids, in which the inset is the detail of the chip resistors. The background region is uniformly divided into square grids with ${\rm{\Delta }}x={\rm{\Delta }}y=2\,{\rm{cm}}$. It is seen that the device is composed of eight triangular regions, in which each triangular region is divided into six layers. For Element I, we choose ${\rm{\Delta }}{x}_{{\rm{I}}}=0.83\,{\rm{cm}}$ and ${\rm{\Delta }}{y}_{{\rm{I}}}=2\,{\rm{cm}}$. For Element II, we choose ${\rm{\Delta }}{x}_{{\rm{II}}}=2\,{\rm{cm}}$ and ${\rm{\Delta }}{y}_{{\rm{II}}}=0.83\,{\rm{cm}}$. The background resistors are chosen as *R*_*x*_ = *R*_*y*_ = 1/(σ_0_*h*) = 51 kΩ. Accordingly, we can obtain *R*_A*x*_ = 390 kΩ, *R*_A*y*_ = 2.4 MΩ, *R*_B*x*_ = 1.2 kΩ, *R*_B*y*_ = 6.2 kΩ for Element I, and *R*_A*x*_ = 510 kΩ, *R*_A*y*_ = 91 kΩ, *R*_B*x*_ = 30 kΩ, *R*_B*y*_ = 5.1 kΩ for Element II, respectively. All these resistors are commercial metal film resistors with an accuracy of 1%. To achieve optically controlled performance, we added light-dependent resistors in parallel to the resistors *R*_B*x*_ of Element II, which is illustrated with green solid lines in the top and bottom triangular regions of Fig. 3(a). The photograph of the fabricated light-programmable meta-device is demonstrated in Fig. S1 (see Supplemental Material).

For reference, we also fabricated a DC cloak as shown in Fig. S2 (see Supplemental Material), which is the same as the structure shown in Fig. 3(a) except that the photoresistors are removed. The simulation result of the reference DC cloak is shown in Fig. 3(b). It is clear shown that the planar potential lines outside the cloak restore exactly to those in homogenous space, which renders the central part invisible to the outside observer. The measured result for the fabricated reference DC cloak is presented in Fig. 3(c), demonstrating excellent cloaking performance. Comparison of Fig. 3(b,c) shows a good agreement between experiment and simulation both inside the cloaking shell and outside the cloak.

**Figure 4:**
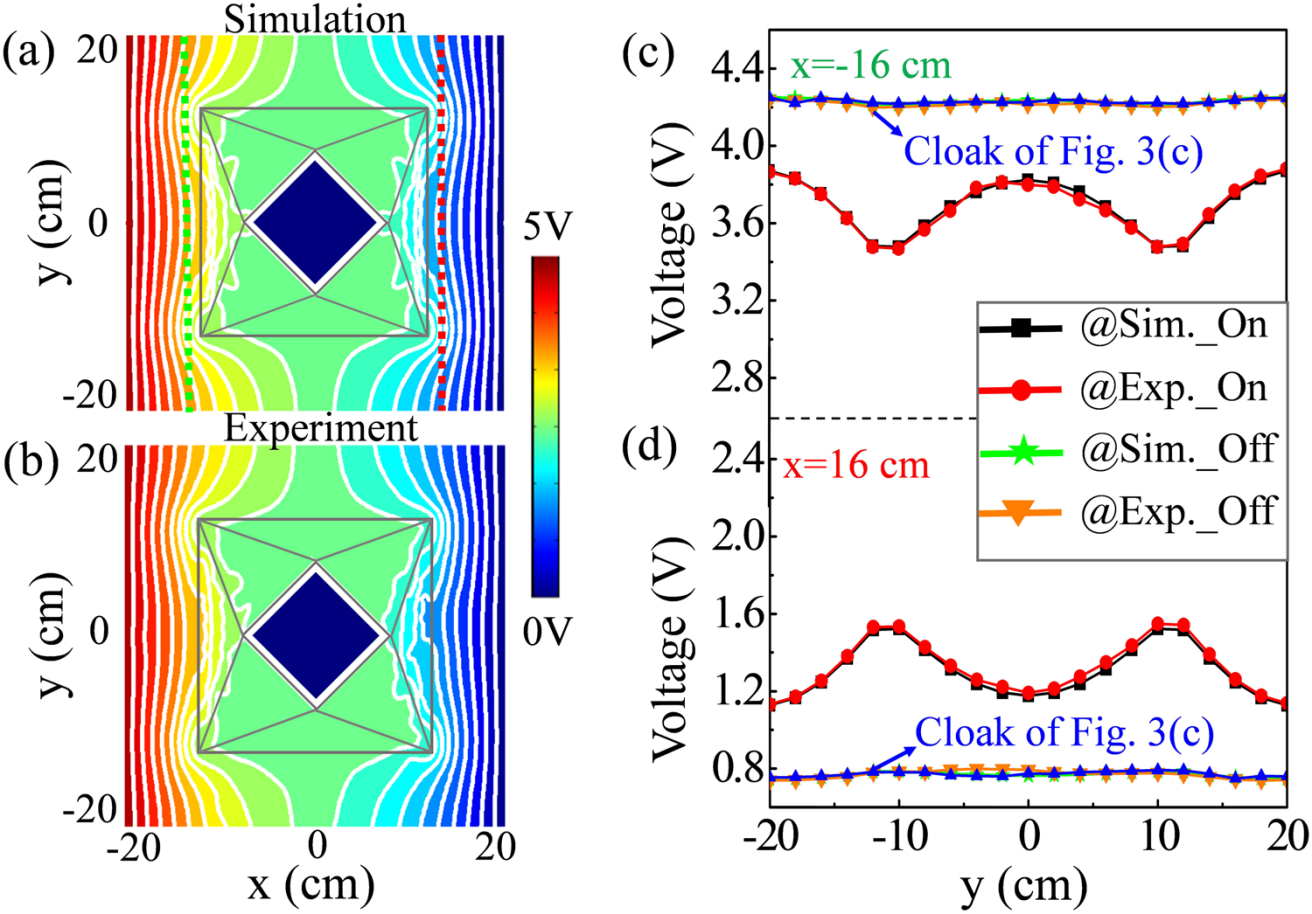
(**a**) Simulated voltage distribution of the meta-device when the whole device is exposed to bright field. (**b**) Measured voltage distribution corresponding to (**a**). (**c**,**d**) The simulated and measured voltage comparison along the observation lines *x* = −16 cm (the green dotted line in (**a**)) and *x* = 16 cm (the red dotted line in (**a**)), respectively. Equipotential lines are represented with white color in the panel.

Next, we examine the performance of the proposed light-controlled meta-device, in which light-dependent resistors are added in parallel to the resistors *R*_B*x*_ of Element II. The real-measured value of photoresistor is 30 MΩ in dark field and 0.5 kΩ in bright field. Since *R*_B*x*_ = 30 kΩ, we obtain ${R^{\prime} }_{{\rm{B}}x}\approx {R}_{{\rm{B}}x}$ in dark field and ${R^{\prime} }_{{\rm{B}}x}\approx {R}_{L}$ in bright field. Therefore, the device acts as an invisibility cloak in dark filed and disguises as different objects in bright field. We first examine the performance of the meta-device in bright field. Figure 4(a) shows the simulated voltage distribution based on the real-measured resistor value (*R*_*L*_ = 0.5 kΩ). Figure 4(b) shows the measured voltage distribution of the meta-device in bright field. A careful examination of Fig. 4(a,b) shows excellent agreement between experiment and simulation. Outside the meta-device, the potential distribution and equipotential lines are exactly the same as those of two virtual triangular insulators, as shown in Fig. 2(d). Obviously, the proposed meta-device acts as an illusion device in bright field. Comparing Fig. 4(a,b), a very small discrepancy is observed in the shell region, which is mainly attributed to the disturbed value of photoresistor.

We then examine the performance of the proposed meta-device in dark field. In order to make sure the measurement carried out in complete darkness, the whole device is covered by an opaque material. In this case, it is not convenient to measure the voltage of each node. However, the performance can be evaluated by measuring the voltage distribution along the observation lines (shown as green and red dotted lines in Fig. 4(a)) near the device. The simulated and measured results are shown in Fig. 4(c,d), which correspond to the voltage distribution at the left observation line (where *x* = −16 cm) and the right observation line (where *x* = 16 cm), respectively. It is clear that the measurement agrees very well with simulation, which validates our design scheme. As expected, the meta-device acts as an illusion device in bright field and becomes an invisibility cloak in dark field. The response time does not exceed 0.2 seconds when the meta-device is switched between cloaking and illusion.

## Discussion

**Figure 5:**
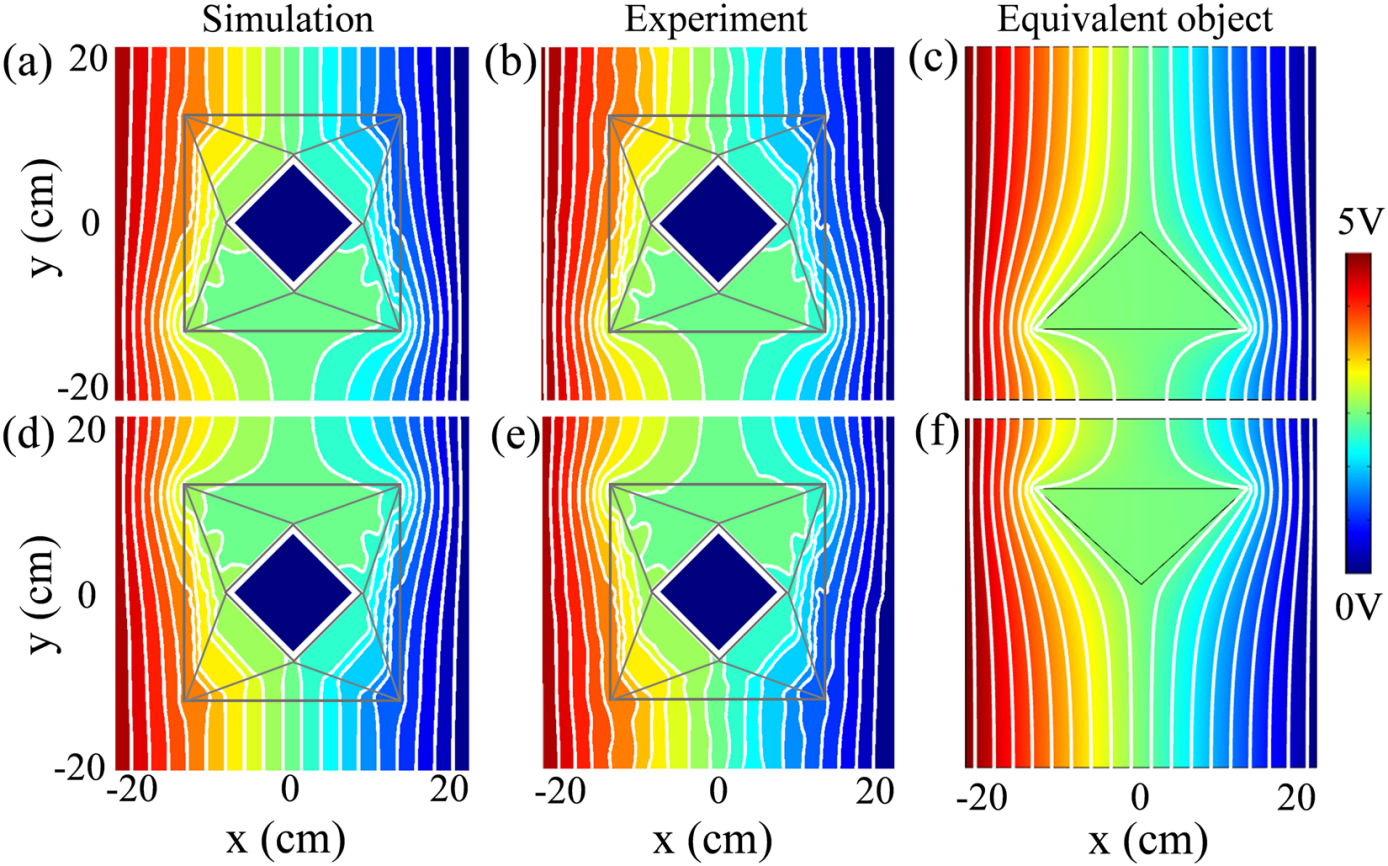
Demonstration of a partial illusion when a part of the device is exposed to bright field. (**a**) Simulated voltage distribution when the lower part of the meta-device is illuminated. (**b**) Measured voltage distribution corresponding to (a). (**c**) Simulated voltage distribution of the equivalent object corresponding to (a). (**d**) Simulated voltage distribution when the upper part of the meta-device is illuminated. (**e**) Measured voltage distribution corresponding to (d). (**f**) Simulated voltage distribution of the equivalent object corresponding to (d). Equipotential lines are represented with white color in the panel.

We demonstrate a partial illusion when a part of the meta-device is exposed to bright field, as shown in Fig. 5. When the lower part of the device is illuminated, Fig. 5(a,b) show the simulated and measured voltage distributions, respectively, which are in good agreement with the equivalent object in Fig. 5(c). In contrast, when the upper part of the device is illuminated, Fig. 5(d,e) show the simulated and measured voltage distributions, respectively, which are also in good agreement with the equivalent object in Fig. 5(f). Obviously, numerical simulations and measurement data have excellent agreement, which demonstrates the controllable and flexible property of the proposed scheme.

In summary, we have proposed a light-programmable meta-device, which shows a multifunction of cloak, full illusion, and partial illusion that is tunable through light illumination. Our scheme requires only four kinds of natural bulk materials with homogeneous parameters throughout, and thus avoids the problems present in previous proposals, such as extreme parameters (inhomogeneous and singular) and discretized approximation. We carried out the proof-of-concept experiment using a resistor-network, with only eight kinds of resistors. The results are in excellent agreement with numerical simulations. We remark that the proposed scheme is not only valid for DC field but also for other fields governed by Laplace equation.

## Methods

### Simulation

We carried out the simulation based on the finite element method (FEM). In our design, we choose *r*_0_ = 1 cm, *r*_1_ = 7 cm, and *r*_2_ = 17 cm. The background has a conductivity of σ  = 1 S/m. In the simulation setup, the computational domain is a square with a side length of 40 cm. The left and right sides of the computational domain are set as fixed voltage with 5 V and 0 V, respectively. The top and bottom sides have insulation boundary conditions. The material of the cloaking object in the central region is copper.

### Fabrication

For Reference, we first fabricated a DC cloak based on the analogy between conducting materials and resistor networks. To make a resistor network equivalent to the continuous conducting material with the thickness *h*, we discretize the continuous material using the Cartesian grids. According to Ohm’s law, each elementary cell in the grid can be implemented by two resistors. All these resistors are commercial metal film resistors with an accuracy of 1%. For the realization of the proposed light-controlled meta-device by the circuit theory, light-dependent resistors are added in certain regions.

### Experiment

In the experimental setup, a DC power supply with 5 V magnitude is used as the source, and the voltage is measured using a 4.5-digit Multimeter.

## Electronic supplementary material

Supplementary Information
