# Supplementary material for: Light-programmable manipulation of DC field in Laplacian Meta-devices
Source: Sci Rep. 2018 Aug 15;8:12208. doi: 10.1038/s41598-018-30612-5 (PMC6093913; doi:10.1038/s41598-018-30612-5)
Supplement: Supplementary file 1 — Supplementary Information [file 41598_2018_30612_MOESM1_ESM.pdf]

Supplementary Information

Light-programmable manipulation of DC field in Laplacian Meta-devices

Tiancheng Han[1,*], Yuexia Liu[1], Lei Liu[1], Jin Qin[1], Ying Li[2], Jiayu Bao[1], Dongyuan Ni[1], and Cheng-Wei Qiu[2,#]

[1]School of Physical Science and Technology, Southwest University, Chongqing 400715, China.

[2]Department of Electrical and Computer Engineering, National University of Singapore, 4 Engineering Drive 3, Republic of Singapore.

Corresponding authors: *tchan123@swu.edu.cn; #chengwei.qiu@nus.edu.sg

1. The photograph of the fabricated devices

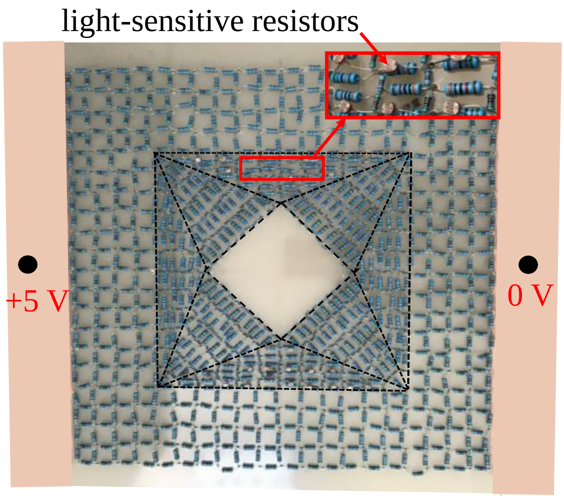

Figure S1. The photograph of the fabricated light-programmable meta-device.

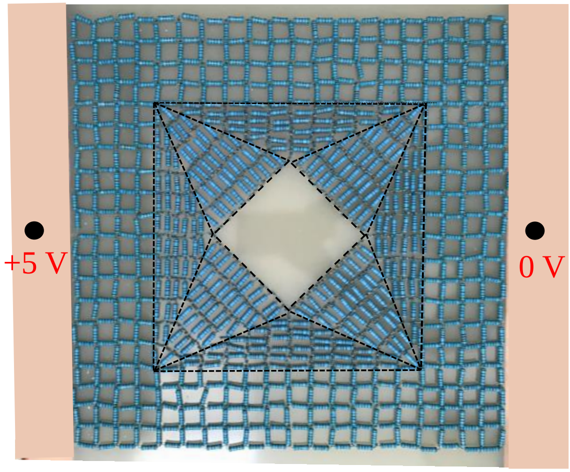

Figure S2. The photograph of the fabricated reference DC cloak.

2. The possible realization of 3D light-programmable meta-device

A bulk conducting materials can be divided into blocks using the rectangular grids, and each block can be mimicked using a 3D resistor networks. A 3D unit cell for the resistor networks is shown in Fig. S3(a). To achieve optically controlled performance, light-dependent resistors may be added in parallel to the resistors R_Z of a certain region, which is illustrated in Fig. S3(b).

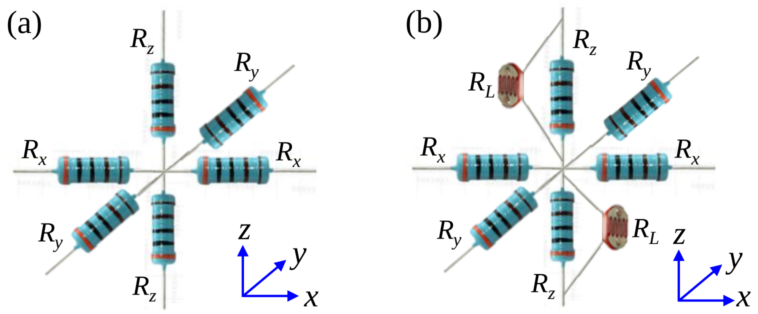

Figure S3. (a) A 3D unit cell for the resistor networks. (b) Light-dependent resistors in parallel to the resistors R_z.
